# From top to bottom: Do Lake Trout diversify along a depth gradient in Great Bear Lake, NT, Canada?

**DOI:** 10.1371/journal.pone.0193925

**Published:** 2018-03-22

**Authors:** Louise Chavarie, Kimberly L. Howland, Les N. Harris, Michael J. Hansen, William J. Harford, Colin P. Gallagher, Shauna M. Baillie, Brendan Malley, William M. Tonn, Andrew M. Muir, Charles C. Krueger

**Affiliations:** 1 Department of Fisheries and Wildlife, Center for Systems Integration and Sustainability, Michigan State University, East Lansing, MI, United States of America; 2 Fisheries and Oceans Canada, Winnipeg, Canada; 3 Department of Biological Sciences, University of Alberta, Edmonton, Canada; 4 U.S. Geological Survey, Hammond Bay Biological Station, Millersburg, MI, United States of America; 5 Cooperative Institute of Marine & Atmospheric Studies, University of Miami, Miami, FL, United States of America; 6 Department of Biology, Dalhousie University, Halifax, Canada; 7 Great Lakes Fishery Commission, Ann Arbor, MI, United States of America; Southwest University, CHINA

## Abstract

Depth is usually considered the main driver of Lake Trout intraspecific diversity across lakes in North America. Given that Great Bear Lake is one of the largest and deepest freshwater systems in North America, we predicted that Lake Trout intraspecific diversity to be organized along a depth axis within this system. Thus, we investigated whether a deep-water morph of Lake Trout co-existed with four shallow-water morphs previously described in Great Bear Lake. Morphology, neutral genetic variation, isotopic niches, and life-history traits of Lake Trout across depths (0–150 m) were compared among morphs. Due to the propensity of Lake Trout with high levels of morphological diversity to occupy multiple habitat niches, a novel multivariate grouping method using a suite of composite variables was applied in addition to two other commonly used grouping methods to classify individuals. Depth alone did not explain Lake Trout diversity in Great Bear Lake; a distinct fifth deep-water morph was not found. Rather, Lake Trout diversity followed an ecological continuum, with some evidence for adaptation to local conditions in deep-water habitat. Overall, trout caught from deep-water showed low levels of genetic and phenotypic differentiation from shallow-water trout, and displayed higher lipid content (C:N ratio) and occupied a higher trophic level that suggested an potential increase of piscivory (including cannibalism) than the previously described four morphs. Why phenotypic divergence between shallow- and deep-water Lake Trout was low is unknown, especially when the potential for phenotypic variation should be high in deep and large Great Bear Lake. Given that variation in complexity of freshwater environments has dramatic consequences for divergence, variation in the complexity in Great Bear Lake (i.e., shallow being more complex than deep), may explain the observed dichotomy in the expression of intraspecific phenotypic diversity between shallow- vs. deep-water habitats. The ambiguity surrounding mechanisms driving divergence of Lake Trout in Great Bear Lake should be seen as reflective of the highly variable nature of ecological opportunity and divergent natural selection itself.

## Introduction

Processes of diversification within and among species are variable, ranging from micro- to macro-evolution, with divergence primarily occurring in allopatry (non-overlapping geographic areas), and to a lesser degree in sympatry (without geographical separation), peripatry (an isolated peripheral geographic area), or parapatry (adjacent geographic area). Diversification patterns can involve coevolution, or parallel, convergent, or divergent evolution, with the processes varying within and across taxa. Trying to unravel these complexities has inspired biologists for decades to investigate population diversification, local adaptation, and speciation [1–4].

Patterns of diversity expressed by freshwater fishes have been of particular interest and often show phenotypic-environmental associations. The complexity (e.g., variable bathymetry) and isolation (e.g., land-water boundaries, glacial history) of freshwater habitats are often linked to phenotypic diversification within a species rather than convergence to a single phenotype [5–7]. Indeed, strong links between ecological and evolutionary processes, through phenotypic plasticity and adaptive evolution, predict the exploitation of differential resources in novel discrete niches by individuals in complex habitats [8, 9]. Within lacustrine systems, however, divergent selection related to resource use within discrete ecological niches is constrained by variation that occurs naturally along littoral, pelagic, and profundal niche axes [10–12].

The profundal zone is regarded as the least favorable habitat within a lake due to low temperature and light conditions, low density of food resources, and sometimes unfavorable water chemistry [13, 14]. Nevertheless, most reported examples of Lake Trout (*Salvelinus namaycush*) intraspecific diversity occur along a depth gradient that includes the profundal zone [15–17]. Lake Trout in large, bathymetrically complex North American lakes, such as Lake Superior, Great Slave Lake, and Lake Mistassini, vary phenotypically as a result of the vertical partitioning of resources and selective pressure(s) that leads to ecological differentiation among morphs [18–20]. Indeed, differences in depth and associated foraging opportunities within these lakes seem to function as “islands” that have promoted and maintained rapid post-glacial adaptive divergence [21–24]. In these North American lakes, Siscowet, humper, and red fin phenotypes are recognized as morphs using deep-water (> 80 m), whereas the lean morph in these large deep lakes occupy shallower habitats (< 80 m) [15].

In general, deep-water morphs have been characterized by deeper bodies and caudal peduncles and higher buoyancy (lipids), in contrast to shallow-water morphs that are characterized by a streamlined body, smaller fins, and a larger head, reflecting locomotion-related traits associated with trophic differentiation [15]. Although the common theme of Lake Trout diversification has focused on isolation by geographic distance in large lakes and within deep waters, examples of diversification in small lakes or shallow-waters habitats have also been documented [25–28]. Overall, these reports confirm high levels of plasticity and diversification in Lake Trout, a characteristic not always recognized [29, 30].

A striking example of Lake Trout intraspecific diversity is the shallow-water (≤ 30 m) morphs in Great Bear Lake, Canada (S1 Fig). Within this zone, four morphs differ in head, body, and fin morphology [25, 31]. Morph 1 is characterized by a small head and intermediate fins. Morph 2 has the largest head and jaws but the smallest fins. Morph 3 has the longest fins and a robust body shape, while Morph 4 has a thick curved lower jaw and smallest caudal peduncle among the morphs. Morph 4 is a specialist in pelagic habitat whereas Morphs 1–3 have more general feeding habits with varying degrees of omnivory along a weak benthic-pelagic shallow-water gradient [27, 32]. Consistent with predictions from trophic polymorphism theory, Morph 1 and 2 differed in adult growth rates, age- and size-at-maturity, and survival rates, whereas Morphs 3 and 4 were intermediate in life-histories [33]. The four morphs were only weakly genetically differentiated and appear to have diverged in sympatry [34].

This pattern of intraspecific diversity, independent of obvious habitat partitioning within the shallow-water zone, is uncommon for polymorphic fishes [35–37]. The pronounced morphological heterogeneity across shallow-water morphs seem somewhat disconnected from expected underlying genetic or trophic mechanisms causing divergence. Lake Trout differentiation within Great Bear Lake, however, could be associated with a depth gradient but has never been investigated. Great Bear Lake is one of the largest and deepest freshwater lakes in North America (surface area = 31 790 km2, maximum depth = 446 m; [38]. Consequently, Great Bear Lake should be sufficiently complex to evolve and sustain diversity across a vertical resource-axis (i.e., niche availability and niche discordance) [39].

To explore the full extent of Lake Trout intraspecific diversity within Great Bear Lake, we investigated whether a deep-water morph co-exists along with four shallow-water morphs previously described. We compared morphology, neutral genetic diversity, trophic ecology, and life-history of Lake Trout caught from depths up to 150 m within Great Bear Lake. We investigated whether Lake Trout genetic and phenotypic variation were partitioned along a depth gradient consistent with Lake Trout differentiation elsewhere. Despite being one of the world’s major freshwater bodies, this study is the first to report on Lake Trout in the deep-water habitat of Great Bear Lake.

## Materials and methods

### Study area and data collection

Great Bear Lake is an oligotrophic Arctic freshwater system, in northeastern Northwest Territories (N66° 06’ W120° 35’) [40]. Great Bear Lake is divided into five semi-isolated ʺarmsʺ. For this study, Dease Arm, within the southern Arctic ecozone along the northern shore of Great Bear Lake, was sampled from mid-July to mid-August 2015. By focusing on one arm and year, we aimed to focus on Lake Trout variation expressed through a cline of depth, while minimizing spatial and temporal variations [6, 8, 41, 42]. This protocol was approved by Department of Fisheries and Ocean Canada, Freshwater Institute Animal Care Committee Science Laboratories (FWI-ACC-2015-036). Once the fish are brought into the boat (from net) they are either already dead or are immediately euthanized by a hit in the head.

Multi-mesh gill nets (12.7 to 140 mm stretch mesh) were set with a typical soak time of 24 hours. Sampling locations were distributed among random depth-stratified sites. Three depth zones were defined: 1) shallow (0–20 m), 2) intermediate (21–50), and 3) profundal zone (51–150) based on productivity levels and the putative deep-water Lake Trout morph distribution [20, 40]. At each sampling station, nets were set on the bottom (0–20 m, 21–50 m, 51–150 m), mid-water (21–50 m, 51–150 m), and just below the surface (0–20 m, 21–50 m). To increase sample size, catch from all meshes and nets were combined when they were defined by depth zone as a categorical variable. For each depth zone, catch-per-unit-effort was calculated as the number of fish caught per 100 m of gill net per 24 hours. Seven, nine, and 10 nets were recorded for 0–20 m, 21–50 m, and 51–150 m, respectively. CPUE data were log_10_ transformed and an Analysis of Variance was used to evaluate if CPUE was significantly different among three depth strata. Lake Trout CPUE did not differ among the three depth strata (F_2,23_ = 0.12, p = 0.89) (S2 Fig).

A lateral (left side) full-body digital image was taken of each fish, with extended caudal, pelvic, and pectoral fins and pinned dorsal and anal fins [43]. For each fish caught, tissues, structures, and traits were sampled for determination of biological characteristics related to life-history, including otoliths, fork length, somatic weight, sex, and stage of maturity (i.e., juvenile, mature, and resting categories; see [25]. Lake Trout from Great Bear Lake do not display sexual differences in morphology and life history [25, 44]; thus, sexes were pooled. A dorsal muscle sample was removed and frozen at -20 °C for stable isotope analysis and a fin clip was stored in 95% non-denatured ethanol for genetic analysis.

### Assignment to groups: Depth, morphology, and composite groups

Due to the propensity of Lake Trout with high levels of morphological diversity to occupy multiple habitat niches, classification of individuals can be challenging [22, 27, 45]. Lake Trout can display variation as a cline rather than strong discontinuity among morphs [22, 45]; thus, our grouping method aimed to accommodate the potential for a gradient of phenotypic variation. To account for all variability expressed among Lake Trout from sampling depths of 0–150 m within Great Bear Lake, assignment of individual Lake Trout to groups was based on three separate independent procedures. We used two grouping methods that are commonly used in intraspecific diversity delineation and a novel one (S3 Fig). Grouping was based on three procedures, using either 1) depth zone, 2) morphology, and 3) a suite of composite variables, including depth (as a continuous variable) and morphology, and also genetics, stable isotopes, and life history traits (S3 Fig).

The first assignment procedure grouped fish by depth-at-capture, using a categorical depth strata: 1) shallow (0–20 m), 2) intermediate (21–50 m), and 3) profundal zone (51–150 m). Categorical classification by depth has been used previously due to the importance of depth as a driver of Lake Trout diversification (see [22]). Morphological, genetic, isotopic, and life history variables were then compared among groups based on depth-at-capture grouping procedure.

The second approach grouped individuals based on morphological data, using the R statistical software package FactoMineR, a hierarchical clustering analysis based on principal components to determine group membership of individuals [46]. Morphological data with principal component and cluster analysis has been commonly used to determine the number of Lake Trout groups co-existing within a system [18, 19, 21, 25, 31]. Morphological data were represented by first principal component (PC1) scores from principal component analyses (PCA) of body shape, head shape, and linear measurements of 130 Lake Trout, using Integrated Morphometrics Programs (IMP) (description below). Morphology, genetics, isotopes, and life history were then compared among groups defined by morphology procedure.

The third approach, termed here as “composite”, assigned individuals to groups based on all collected ecological data, using FactoMineR to assign individuals to groups. Habitat was measured as depth-at-capture (continous variable). Morphological data included first two principal components of PCA for each morphological variable to capture the full extent of variation (i.e., body shape, head shape, and linear measurements). Genetic data included the first two principal components of a PCA using 21 microsatellite loci, and trophic data included stable isotopes (muscle δ^13^C and δ^15^N). Finally, life-history data included parameters obtained from back-calculated otolith data: von Bertalanffy adult growth parameter, juvenile growth rate, and maximum adult length. These composite data were available for 105 of the 130 Lake Trout. Morphology, genetics, isotope data, and life history were then compared among groups defined by the composite procedure. Details on morphology, stable isotopes, life-history, and genetic analyses are provided below.

### Morphology

Analyses of digital images, combining classical with geometric morphometrics, were performed only on adult Lake Trout (based on gonad devlopment and length over 450 mm, see [25]) due to the difficulty of classifying juveniles into morphs [18, 19, 25]. Twenty three landmarks (pre-determined homologous points) were selected to measure body shape and fourteen linear measurements were selected based on their relationship to foraging (e.g., jaw size) and swimming (e.g., fin lengths and caudal peduncle depth) [47–49]. Landmarks used in this study were comparable to those used previously for assessing Lake Trout intraspecific diversity in Great Bear Lake (see [25, 31]). We also used 20 semi-landmarks to measure variation in head shape that were not well captured by landmarks. Semi-landmarks are non-homologous points that can be used to capture and analyze shape information on curved areas of lacking distinct landmarks [50, 51].

Landmarks and semi-landmarks were digitized in x and y coordinates using TPSDig2 software (http://life.bio.sunysb.edu/morph). Subsequently, digitized landmarks and semi-landmarks were processed in a series of Integrated Morphometrics Programs (IMP) (http://www2.canisius.edu/;sheets/morphsoft) (methods and programs described in [51]. All morphological measurements were size-free, using centroid sizes or residuals from regressions on standard length for linear measurements [51]. Principal component analyses (PCA) of morphological data (body and head shape used PCAGEN; IMP software, and linear measurements using PC-ORD) for both 130 and 105 individuals were performed on all morphological data to capture the maximum amount of variation with the fewest number of variables for subsequent grouping analysis (procedures described in [25].

#### Morphological characteristics among groups within each grouping procedure

To visualize morphological variations among groups, PCAs of the 130 and 105 individuals used above in FactoMineR, were displayed with classified individuals from each grouping procedure assignments, groups were defined with confidence ellipse. To assess the validity of group assignments in these morphological variations, canonical variate analyses (CVA) followed by Jackknife validation procedures were used to test how well linear measurements, body and head shape grouped individuals with CVAGen V. 8 from the IMP software (http://www3.canisius.edu/~sheets/).

### Genetic data

Genomic DNA was extracted from fin tissue using Qiagen DNeasy Extraction Kits (Qiagen, Inc., Valencia, California) following the manufacturer's protocols. Twenty-one microsatellite loci were amplified in four multiplexes [34]. PCR products were run on an ABI 3130xl Genetic Analyzer (Applied Biosystems, Foster City, California) using the LIZ 600 size standard and all allelic data were edited and scored by eye using GeneMapper (version 4.0, Applied Biosystems).

#### Genetic characteristics among groups within each grouping procedure

**V**ariation at microsatellite loci was used to determine if genetic differentiation could be resolved among Lake Trout groups established by the three grouping procedures (depth, morphology, and composite groups; S3 Fig). The program Microchecker v.2.2.3 [52] was used to test each locus for the presence of genotyping errors due to null alleles and allelic dropout. Descriptive statistics of genetic variation (number of alleles [N_A_], expected heterozygosity [H_E_; Nei’s unbiased gene diversity], observed heterozygosity [H_O_], and the fixation index [F_IS_]) within groups for each grouping procedure (i.e., depth, morphological and composite) were compiled using the ‘diveRsity’ package in program R [53]. Allelic richness (A_R_) and private allelic richness (PA_R_) were calculated using HP-RARE [54]. Departures from Hardy-Weinberg equilibrium and linkage disequilibrium were evaluated using the program GENEPOP v. 4.0 [55]. Tests involving simultaneous comparisons were evaluated with a nominal α of 0.05 and then with an adjusted α obtained via the false discovery rate procedure [56], as suggested by Narum [57].

Genetic structure was examined among Lake Trout groups using several different methods. Global estimates of F_ST_ (i.e., theta [θ]) [58] for each grouping procedure were generated in FSTAT and 95% confidence intervals (CIs) of the estimates were calculated following 10 000 permutations. Pairwise estimates of F_ST_ between groups within a procedure were calculated in ARLEQUIN v. 3.1 [59], significance was tested following 10 000 individual permutations. We used the hierarchical Bayesian clustering program STRUCTURE v. 2.3 [60] to identify potentially distinct genetic clusters within groups (e.g., by depth, morphology, or composite). Simulations were performed varying K from 1 to 10, with 20 iterations per value of K. Each run incorporated a burn-in of 500 000 iterations followed by 500 000 Markov chain–Monte Carlo (MCMC) iterations. We assumed an admixture model, correlated allelic frequencies, and grouping based on depth, morphology, or composite, as location information as priors [61]. To infer the most likely number of clusters, we used STRUCTURE HARVESTER v. 0.6.91 [62], a program that combines the results of independent runs and compiles the results based on lnP(D) and the *post hoc* ΔK statistic of Evanno et al. [63]. The program CLUMPP v. 1.1 [64] was used (under the LargeKGreedy algorithm) to determine alignment of replicate runs and admixture plots were visualized using DISTRUCT v.1.1 [65].

### Isotopes

Samples analyzed for isotopes were freeze dried, ground to a fine powder, and weighed. Samples were analyzed using a continuous flow isotope ratio mass spectrometer (Thermo- Delta 5 Plus) equipped with a Costech elemental analyzer at the Department of Fisheries and Ocean’s Freshwater Institute in Winnipeg. Stable isotope results were expressed in delta (δ) notation defined as the deviation from a standard reference material in parts per thousand (‰). δ^13^C results are relative to Vienna Pee Dee Belemnite (VPDB) while δ^15^N results are relative to atmospheric nitrogen and were calculated using equation that follows:

δX=[(Rsample/Rstandard)–1]×1000

where X is ^13^C or ^15^N, Rsample is the ratio (^13^C/^12^C or ^15^N/^14^N) in the sample while Rstandard is the same ratio in the standard. Standard deviations of repeated measurements of certified reference materials (USGS 40 and 41) were <0.1‰ for δ^13^C and <0.16‰ for δ^15^N. The standard deviation of repeated measurements of an in-house standard was <0.1‰ for δ^13^C and for δ^15^N. Data were normalized using Laboratory Information Management System for Light Stable Isotopes (LIMS-LSI) [66]. Due to high C:N ratios (>3.5) indicating high-lipid content, the fish δ^13^C values were lipid-corrected following Post et al. [67].

#### Isotopic characteristics among groups within each grouping procedure

Niche region dimensions of Lake Trout (grouped by depth, morphotype, and composite variables) were obtained using the probabilistic method available in the nicheROVER R library [68]. This approach estimates parameters of the multivariate normal distribution, allowing isotopic niche dimentions to be defined as probability regions in multivariate space. Uncertainty in niche regions is accounted for using a Bayesian inference famework [68]. Ellipses representing 95% probability niche regions were generated using the posterior expectation of the bivariate normal distribution estimated using the Bayesian approach in nicheROVER [68]. C:N ratios were also regressed against δ^13^C (‰) (not lipid-corrected values), individuals were grouped by depth-at-capture to investigate an indirect representation of lipid content (index of buoyancy) according to the depth structure found elsewhere [18–20, 67, 69–71]. A polynomial trend line was fitted for the overall data and was tested for differences from 0.

### Life-history

One otolith from each fish was embedded in epoxy, and a thin transverse section (400 μm) was cut, mounted on a glass slide, polished, and imaged for age and growth assessment [72]. Annuli were counted by two independent readers using criteria described by Casselman and Gunn [73]. Age estimates were used to inform demarcation of growth increments, measured from the nucleus to the maximum ventral radius of the otolith, and radial measurements at each annulus were used to back-calculate length-at-age using the biological intercept back-calculation model [74]. The biological intercept (sagittal otolith radius = 0.137 mm; age-0 Lake Trout length = 21.7 mm; [72] was based on equations describing relationships between length, age in days, and sagittal otolith width of age-0 Lake Trout [75].

Growth in length with age was modeled using two parameterizations of the Von Bertalanffy length-age model, which express growth in terms of five, rather than only three, life history parameters [76, 77]:

$$L_{t}=L_{\infty }\left(1-e^{-K(t-t_{0})}\right)+\varepsilon$$

$$L_{t}=L_{\infty }-\left(L_{\infty }-L_{0}\right)\left(1-e^{-\left(\omega /L_{\infty }\right)\times t}\right)+\varepsilon$$

These length-age models described back-calculated length *L*_*t*_ (mm) at age *t* (years) as a function of age at length = 0 (t0 = years), length at age = 0 (*L*_0_ = mm; length at emergence from the egg), early annual growth rate (ω = L∞ × K = mm/year; [78], instantaneous growth rate (*K* = 1/year) at which *L*_*t*_ approaches the theoretical maximum length (*L*_∞_ = mm), and residual error (*ε*). Parameters were estimated using nonlinear mixed-effect models (package ‘nlme’ in R) [79], with a fixed population effect, random individual effects, and depth zone, morphological, or composite group as a fixed factor, to compare average growth curves among depth, morph, or composite groups [80]. Mixed-effects models described the within-group correlation of longitudinal, auto-correlated, and unbalanced data, such as back-calculated growth histories [81].

#### Life-history characteristics among groups within each grouping procedure

To compare life-history parameters among groups for each grouping procedure (i.e., depth, morphology, and composite), log-likelihoods (*LL*) of models (*i*) with 1–3 fixed growth parameters (*L*_∞_, *K*, *t*_0_) and depth, morph, or composite groups (*df* = number of parameters) were ranked using Akaike’s Information Criterion (*AIC* = −2 × *LL* + 2 × *df*), *AIC* differences (Δ_*i*_ = *AIC*_*i*_ − *AIC*_*min*_), and *AIC* weights ($w_{i}=\frac{e^{\left(-0.5\Delta_{i}\right)}}{\sum_{r=1}^{R}e^{\left(-0.5\Delta_{r}\right)}}$) to express the relative likelihood that a particular model was the best model among those considered [82].

### Phenotypic divergence characteristics among groups within each grouping procedure

Among- (P_ST_) and within-group (*r*ii) phenotypic variance was estimated for morphological traits for each procedure by calculating genetic relationship matrices (R-matrix estimates) in program RMET 5.0 [22, 83, 84]. Phenotypic distances were adjusted for sample size in RMET, and a heritability score of 1.0 was used to provide a conservative estimate for P_ST_ [85, 86]. Because heritability was set to 1.0, our Pst estimates were conservative and prone to false negatives (Type II Errors) [22]. GP_ST_s were either based on the residuals of the leading principal component (PC) axis, combining all traits for body and head shape and linear measurements, or on specific traits, life-history measures (*ω*, K, and *L*_∞_), and C:N ratio (as an indirect measure of lipids and buoyancy).

## Results

### Assignment to groups: Depth, morphology, and composite groups

Based on depth of capture, 130 adult Lake Trout were categorized into three depth-range groups: 44 fish were caught in 0–20 m water, 55 fish were in 21–50 m, and 31 fish in 51–150 m.

Based on the first principal components scores of morphology based on body shape, head shape, and linear measurements, three groups were determined by FactoMineR among the 130 adult Lake Trout (S4 Fig). The three morphs found in this new dataset corresponded to previously described Lake Trout morphs 1, 2, and 3 from Great Bear Lake [25]. From FactoMineR, hierarchical clusters of Lake Trout, overlaid on the first two principal component variables, explained 75.0% for PC1 and 16.1% for PC2 of the morphological variation (S4 Fig).

Based on composite grouping procedure (depth-at-capture, morphology, genetics, isotopes, and life-history), four groups were determined by FactoMineR among 105 Lake Trout (S4 Fig). The first three groups corresponded approximately to morphs previously described by Chavarie et al. [25], identified as Comp 1 (looks like Morph 3), Comp 2 (looks like Morph 1), Comp 3 (looks like Morph 2), and the last group consisted mostly of individuals caught in the deep-water strata identified as Comp 4. This fourth group comprised 24 individuals of the 105 (22.9%), and reflected grouping differences between the two data sets (morphology vs. composite). Another difference between grouping approaches was that 26 of 105 (24.8%) individuals classified as Morph 2 (piscivore) strictly using morphological data were classified as Morph 1 (generalist) using the composite grouping procedure. From FactoMineR, the hierarchical clusters of Lake Trout individuals overlaid on the first two principal components explained 24.6% (PC1) and 18.2% (PC2) of the variation (S4 Fig 4). The two principal component variables from the composite grouping explained a total of 42.8%, considerably less than the morphological grouping procedure (91.1%).

### Morphological characteristics among groups within each grouping procedure

**Fig 1:**
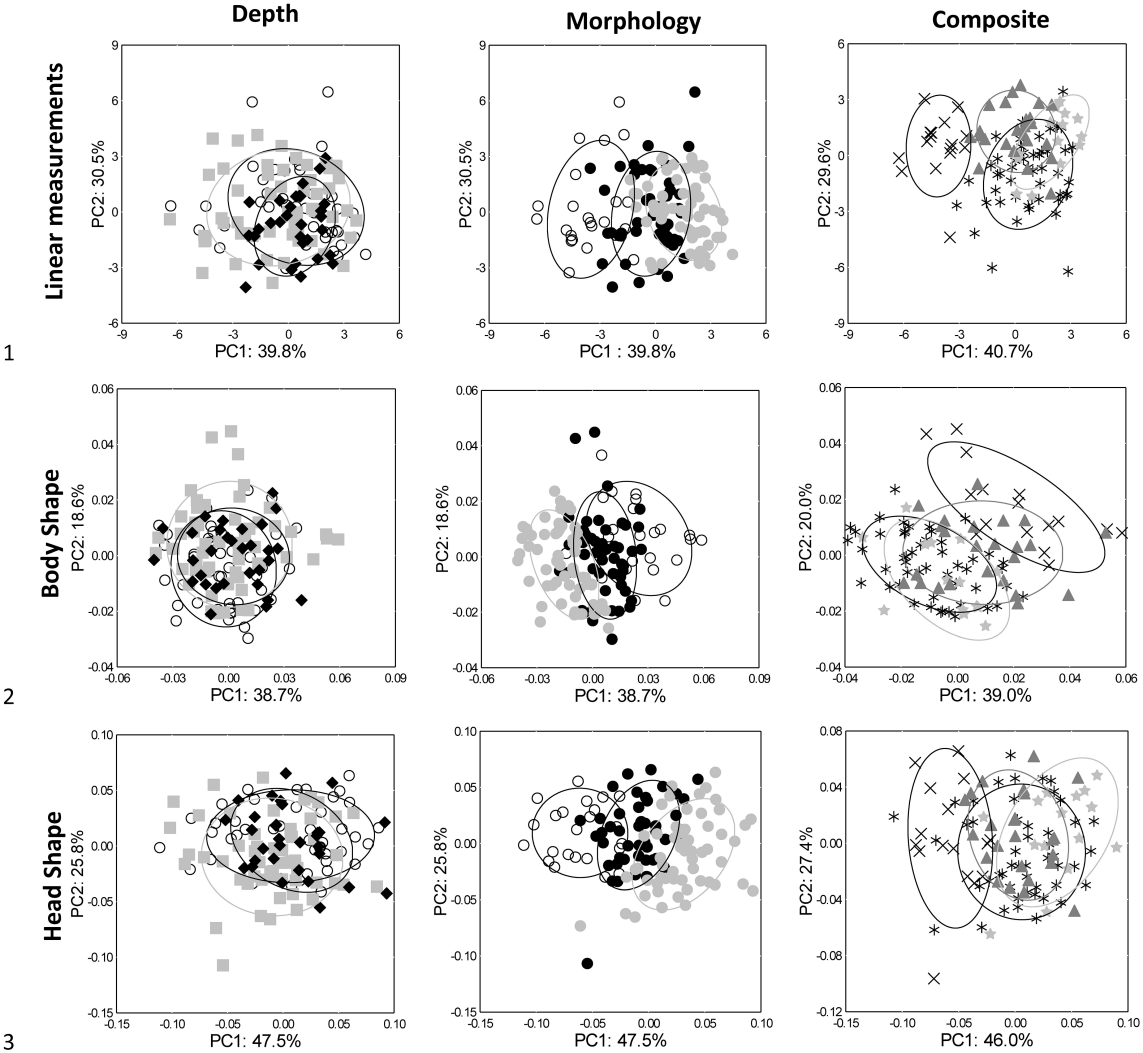
PCA ordinations of Lake Trout linear measurements, body shape, and head shape. PCA ordinations of Lake Trout linear measurements, body shape, and head shape, with percentages representing the variation explained by that component. Groups were identified by three separate procedures using either depth, morphological, or composite data (see text in Methods). Each group is outlined by a 68.3% confidence ellipse. For the depth procedure, groups are represented as follows: open circle = 0–20 m, light grey square = 21–50 m, and black diamond = 51–150 m. For morphological procedure, groups are represented as follows: Morph 1 = white, Morph 2 = black, and Morph 3 = light grey. For composite assignments, groups are represented as follows: x = Comp 1, ⋆ = Comp 2, star = Comp 3, and triangle = Comp 4 (deep-water individuals).

The principal components 1 and 2 for the 130 individuals accounted for 39.8% and 30.5% of the variation in linear measurements, respectively, 38.7% and 18.6% for body shape, and 47.5% and 25.8% for head shape (Fig 1). The PCA from 130 individuals, using classification from depth and morphological grouping assignments, were represented in Fig 1. Nearly the same amount of variation was explained for individuals for the PCA using 105 of the 130 individuals (composite groupings procedures vs. from depth and morphological grouping procedures) (Fig 1). In this case, principal components 1 and 2 accounted, respectively, for 40.7% and 29.6% of the variation in linear measurements, 39.0% and 20.0% for body shape, and 46.0% and 27.4% for head shape (Fig 1).

On the basis of groups established by morphology, CVAs showed group discrimination as follows: body shape CV1: λ = 0.01, CV2: λ = 0.6; *P* < 0.05, and head shape CV1: λ = 0.09, CV2: λ = 0.5; *P* < 0.05 (S5 Fig). Jackknife classification for body shape was 75.6% and 70.0% for head shape. Linear measurement CVA also demonstrated discrimination among morphs with a λ = 0.18, *P* < 0.05 and a jackknife classification of 80%.

On the basis of groups established by composite data (4^th^ group corresponding to individuals caught in deep-water), group discriminations based on morphological characteristics were as follows: body shape CV1: λ = 0.05, CV2: λ = 0.3; *P* < 0.05, and head shape CV1: λ = 0.05, CV2: λ = 0.2; *P* < 0.05 (S5 Fig). However, the jackknife classifications were low with 53.3% correct assignment of individuals based on body shape and 43.8% for head shape. The linear measurement CVA discriminated among composite groups (λ = 0.11, *P* < 0.05) and provided a correct classification of 74.8%.

### Genetic characteristics among groups within each grouping procedure

The locus Smm21 was identified as having null alleles and Sco218 was monomorphic. Both were removed from subsequent analyses. Genetic variation was moderate among individuals and the number of alleles averaged across all loci was 18.2 for depth, 15.4 for morphological, and 12.9 for composite groupings (Table 1). Other descriptive statistics of genetic variation were relatively similar within and among grouping procedures (Table 1). Deviations from Hardy-Weinberg equilibrium expectations were detected in twelve of 63 comparisons for depth (3 groups x 21 loci), six of 63 comparisons for morphology (3 groups x 21 loci), and four of 84 comparisons (4 groups x 21 loci) for composite grouping procedure after false discovery rate adjustments of alpha. Linkage disequilibrium was detected in 21 of 630 tests for depth, five of 630 tests for morphological, and four of 840 tests for composite groupings subsequent to adjustments of alpha based on the false discovery rate procedure.

**Table 1:** Genetic variation at 21 microsatellite loci among groups of Lake Trout from Great Bear Lake established based on depth strata (≤20 m, 21–50 m, 51–150 m), morphological data (Morph 1, Morph 2, Morph 3), and composite data (Comp 1, Comp 2, Comp 3, Comp 4). Columns indicate the number of alleles N_A_, observed heterozygosity (*H*_O_), expected heterozygosity (*H*_E_), Allelic richness (A_R_), private allelic richness (PA_R_), and the fixation index (F_IS_).

Procedure	Groups	N_A_	H_O_	H_E_	A_R_	PA_R_	F_IS_
**Depth Zone**	≤ 20 m	18.19	0.77	0.81	13.63	1.72	0.05
	21–50 m	19.57	0.77	0.81	13.72	1.87	0.05
	51–150 m	15.00	0.76	0.80	12.82	1.35	0.04
**Morphology**	Morph 1	15.62	0.75	0.80	13.16	1.43	0.06
	Morph 2	17.29	0.77	0.81	13.39	1.82	0.04
	Morph 3	13.14	0.77	0.83	13.14	1.68	0.08
**Composite**	Comp 1	11.29	0.79	0.83	11.29	1.00	0.05
	Comp 2	16.33	0.77	0.80	13.35	1.41	0.04
	Comp 3	11.05	0.77	0.81	11.05	1.13	0.04
	Comp 4	12.76	0.75	0.81	12.76	1.28	0.07

Genetic differentiation within each grouping procedure was low. Global estimates of F_ST_ were 0.004 (95% CI = 0.001–0.008) for depth, 0.004 (95% CI = 0.000–0.009) for morphological, and 0.003 (95% CI = 0.000–0.006) for composite groupings (Table 2). Pairwise estimates of F_ST_ within each group were also low (all pairwise values were < 0.01) and only significant in five of a possible 15 comparisons across all grouping procedures subsequent to false discovery rate adjustments of alpha (Table 2).

**Table 2:** Pairwise F_ST_ based on variation at 21 microsatellite loci among Lake Trout from Great Bear Lake grouped by depth strata (≤20 m, 21–50 m, 51–150 m), morphology (Morph 1, Morph 2, Morph 3), and composite data (Comp 1, Comp 2, Comp 3, Comp 4). Significant results are represented as follows: * values are significant at α = 0.027 and 0.020 for 3 and 4 comparisons respectively subsequent to false discovery rate adjustments.

Procedure		Groups		
		≤ 20 m	21–50 m	51–150 m
**Depth Zone**	≤ 20 m			
	21–50 m	0.001		
	51–150 m	0.009*	0.006*	
		Morph 1	Morph 2	Morph 3
**Morphology**	Morph 1			
	Morph 2	0.001		
	Morph 3	0.004	0.007*	
		Comp 1	Comp 2	Comp 3
**Composite**	Comp 1			
	Comp 2	0.007*		
	Comp 3	0.003	0.001	
	Comp 4	0.005	0.008*	0.005

**Fig 2:**
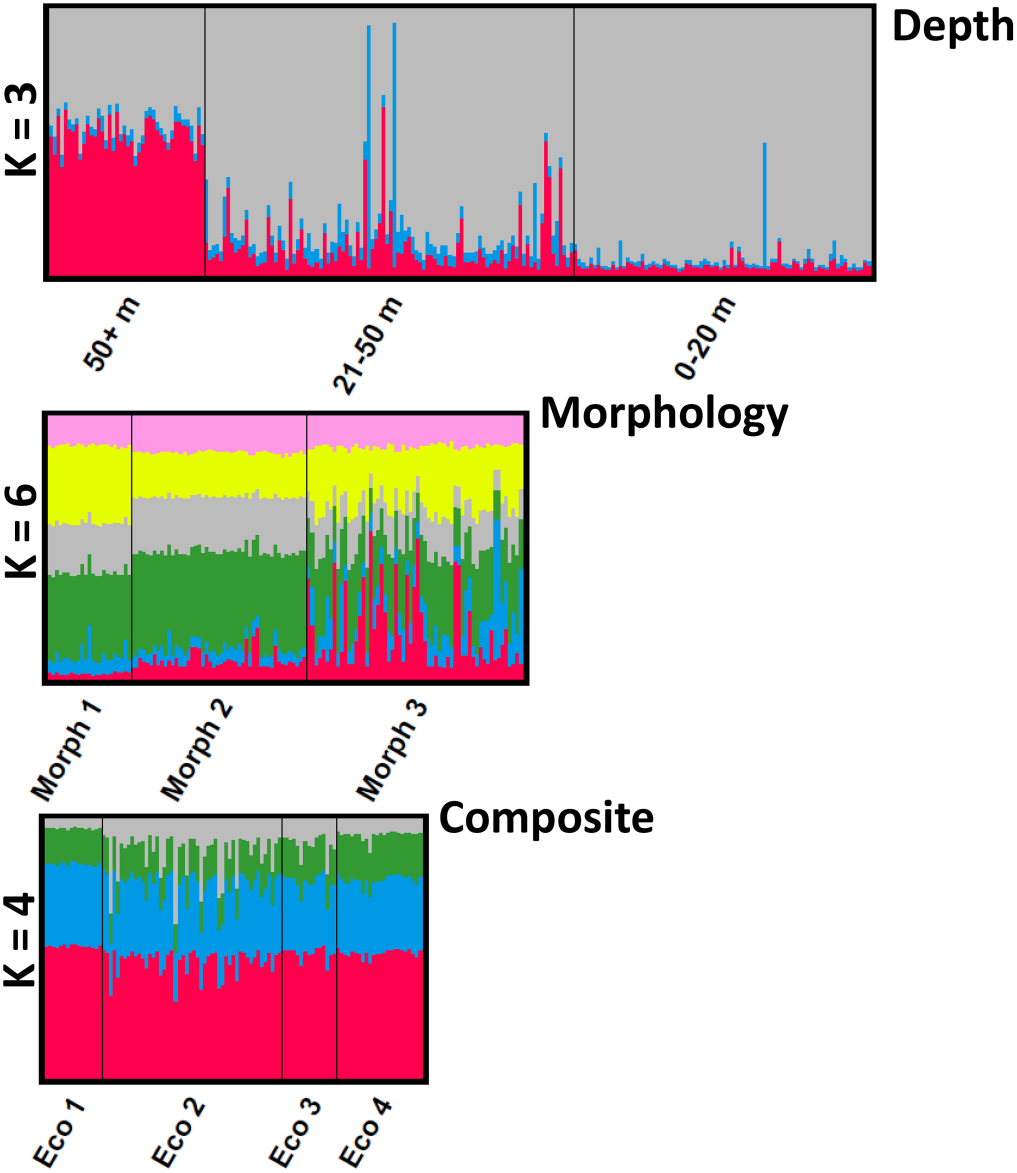
Admixture coefficient plots of the Bayesian clustering analysis for Lake Trout using STRUCTURE. Admixture coefficient plots of the Bayesian clustering analysis for Lake Trout from Great Bear Lake using STRUCTURE. Population structure was examined by groups defined by depth zone (0-20m, 21–50 m, 51–150 m), morphological data (Morph1, Morph 2 and Morph 3), and the composite dataset (Comp 1, Comp 2, Comp 3, and Comp 4). Each individual is represented as a vertical line partitioned into colored segments representative of an individual’s fractional membership in any given cluster (K). The most likely number of genetic clusters based on the ΔK statistic of Evanno et al. [63] was three, six and four for depth, morphology, and composite grouping respectively. The most likely number of clusters based on the traditional statistic mean LnP(K) was K = 1 for each scenario.

Bayesian clustering analyses based on the *post hoc* ΔK statistic of Evanno et al. [63] identified three genetic groups for depth, six genetic groups for the morphological grouping, and four genetic groups for composite grouping (S1 Table). The number of genetic groups inferred based on lnP(D) was one for every grouping procedure. The clearest genetic structure evident based on admixture plots of inferred clusters from ΔK, was the differentiation of the deep-water individuals (51–150 m) from the shallow-water individuals (0–20 m and 21–50 m) for adults (Fig 2). Morph 2 also appeared somewhat differentiated compared to Morphs 1 and 3 in the morphological grouping (Fig 2).

### Isotopic characteristics among groups within each grouping procedure

**Fig 3:**
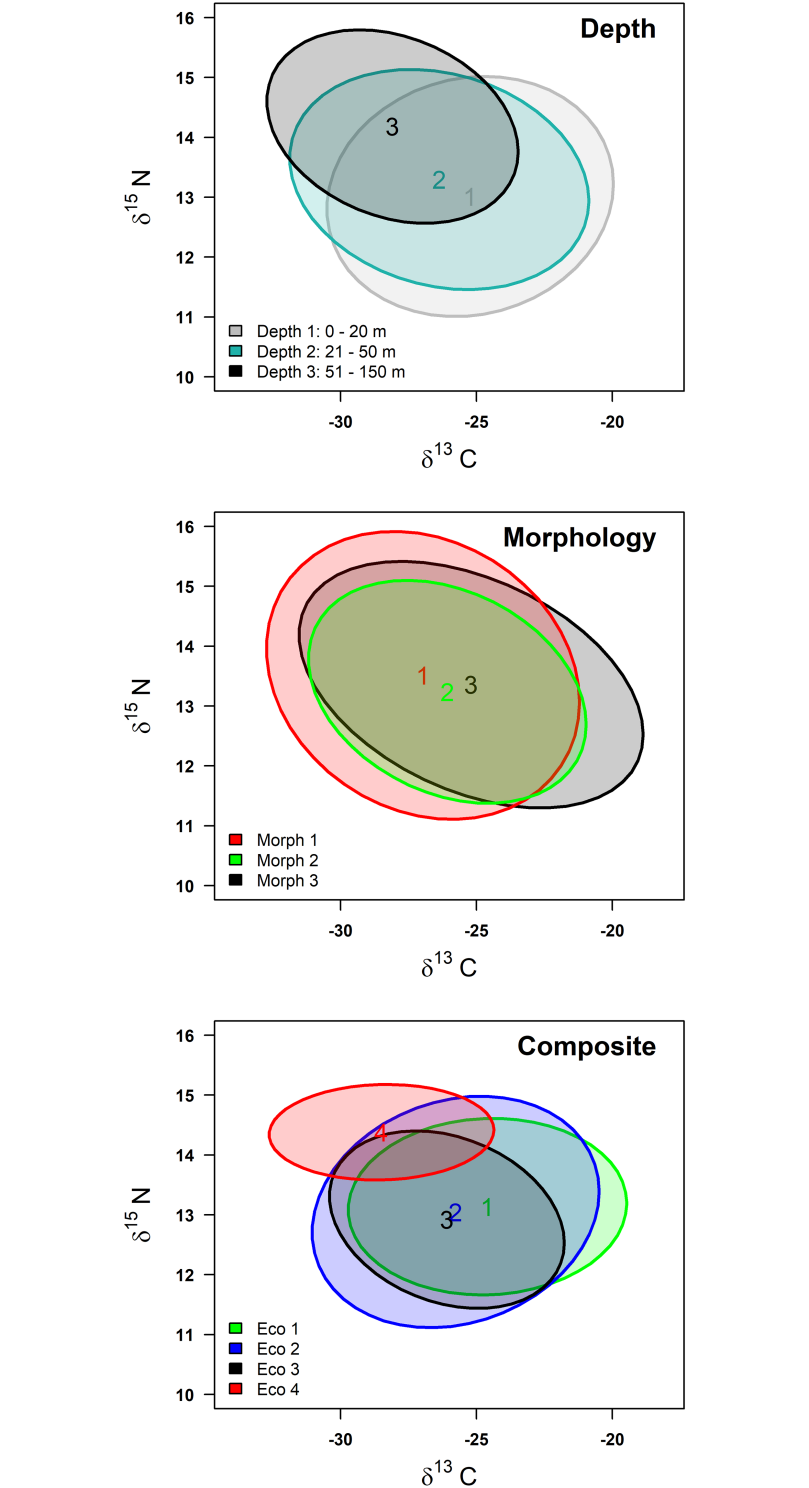
Probabilistic (95%) niche ellipses based on δ^13^C ‰ and δ^15^N ‰ stable isotopes. Probabilistic (95%) niche ellipses based on carbon (δ^13^C ‰) and nitrogen (δ^15^N ‰) stable isotopes for groups of Lake Trout in Great Bear Lake classified by depth zone, morphology, and composite procedures (colors match Fig 2).

**Fig 4:**
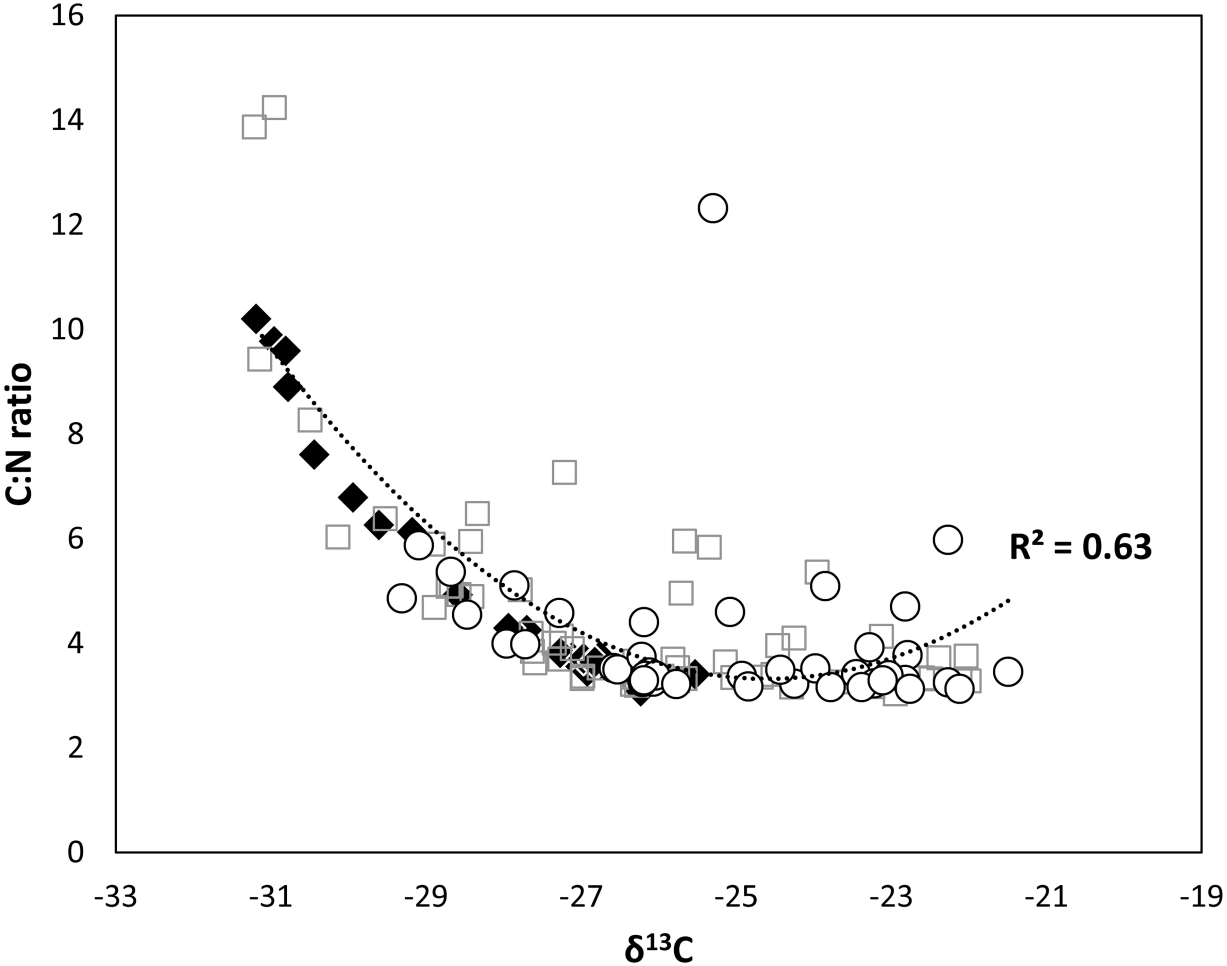
Trend between C:N ratio and δ^13^C (‰) in individual Lake Trout from three depth strata. Trend between C:N ratio and δ^13^C (‰) in individual Lake Trout from Great Bear Lake caught from three depth strata: open circle = 0–20 m, light grey square = 21–50 m, and black diamond = 51–150 m. A polynomial trend line was fitted for the overall data. C:N ratios are an indirect representation of lipid content (index of buoyancy).

The range of stable isotope values was wide among Lake Trout caught among depth zones (Fig 3). For adult Lake Trout, δ^13^C varied from -16.4 ‰ to -27.8 ‰ and δ^15^N ranged from 11.1‰ to 15.3‰. The isospace plot suggested considerable overlap in isotopic niches for all grouping approaches, with differences in niche widths and positions of some deep-water group individuals (Fig 3). Polynomial trend lines had R^2^ of 0.45 for adult Lake Trout (Fig 4), demonstrating an indirect increase of lipid content (index of buoyancy) with depth.

### Life-history characteristics among groups within each grouping procedure

**Fig 5:**
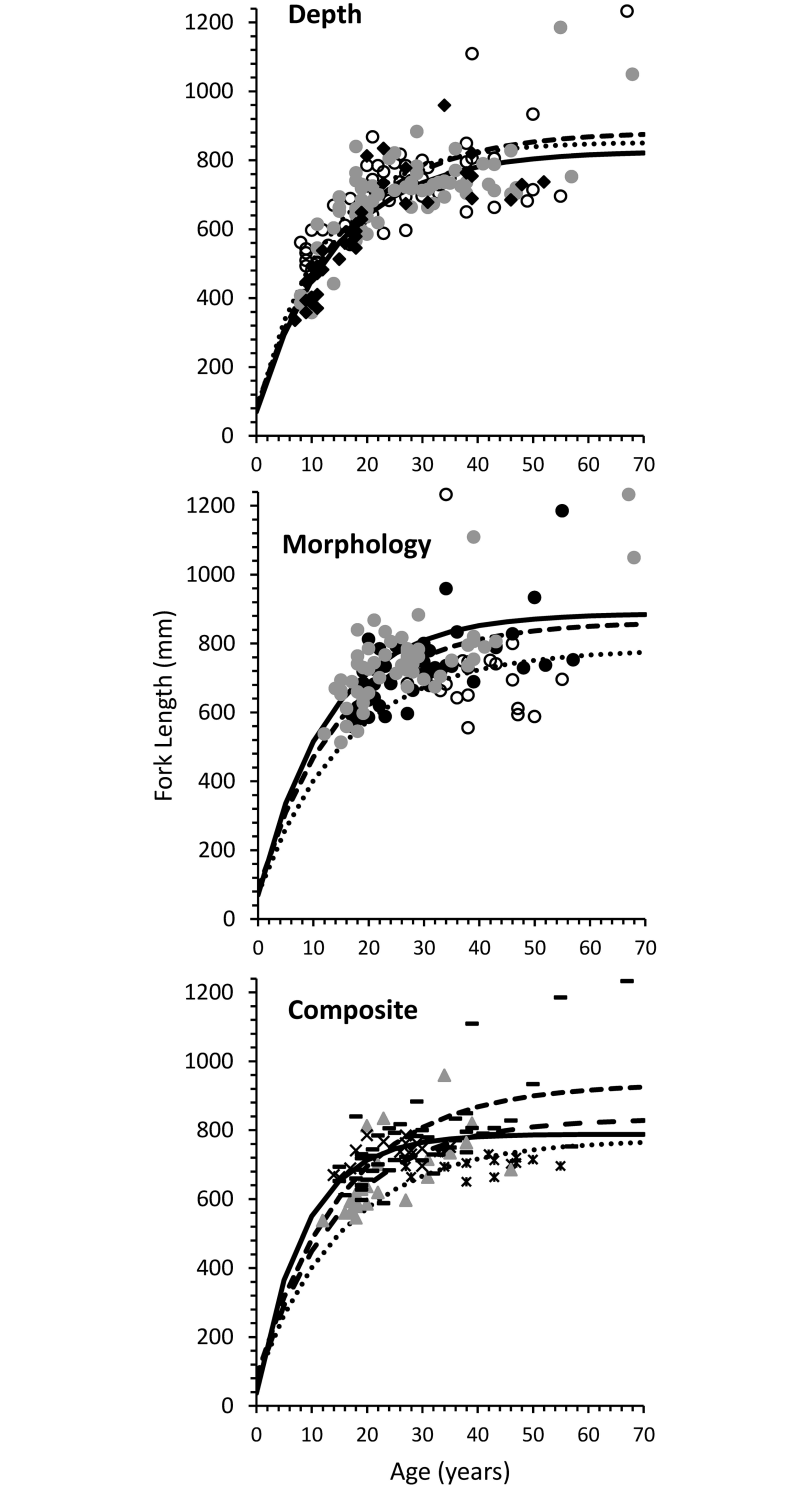
Length at age of Lake Trout captured in three depth zones. Length at age of Lake Trout captured in three depth zones (0−20 m = ○ and dotted line; 21−50 m = gray ○ and dashed line; 51–150 m = ♦ and solid line) and classified into three morphs (Morph 1 = ○ and dotted line; Morph 2 = ● and dashed line; Morph 3 = gray ○ and solid line) and four composite groups (Comp 1 = * and dotted line; Comp 2 = —and dashed line, Comp 3 = **×** and solid line; and Comp 4 = gray ▲ and long-dashed line) in Great Bear Lake.

Growth differed among groups categorized by depth, but growth differences were not clearly attributable to either asymptotic length (*L*_∞_), growth rate (*K*), or age at length = 0 (*t*_0_). The four most likely models of growth variation within depths were 12–35% likely the best model among those considered, without any single model being clearly the best model (S2 Table). Instantaneous growth rate *K* and early growth rate *ω* were highest for trout caught at 0–20 m, whereas asymptotic length *L*_∞_ were highest for the 21–50 m group (Fig 5; S3 Table).

Growth differed among groups based on morphological data. The most likely model of growth variation among morphs was 86% likely the best model, among those considered, and included all three growth parameters (asymptotic length (*L*_∞_), growth rate (*K*), and age at length = 0 (*t*_0_)) (S4 Table). The instantaneous growth rate *K*, the early growth rate *ω*, and the asymptotic length *L*_∞_ were highest for Morph 2 (piscivorous morph) (Fig 5; S5 Table).

Growth differed among groups identified using the composite procedure. The most likely model of growth variation among composite groups was 100% likely the best model, among those considered, and included all three growth parameters (asymptotic length (*L*_∞_), growth rate (*K*), and age at length = 0 (*t*_0_)) (S6 Table). The instantaneous growth rate *K* and early growth rate *ω* were highest for Comp 3, whereas asymptotic length *L*_∞_ was highest for the Comp 2 (Fig 5; S7 Table).

### Phenotypic divergence characteristics among groups within each grouping procedure

**Fig 6:**
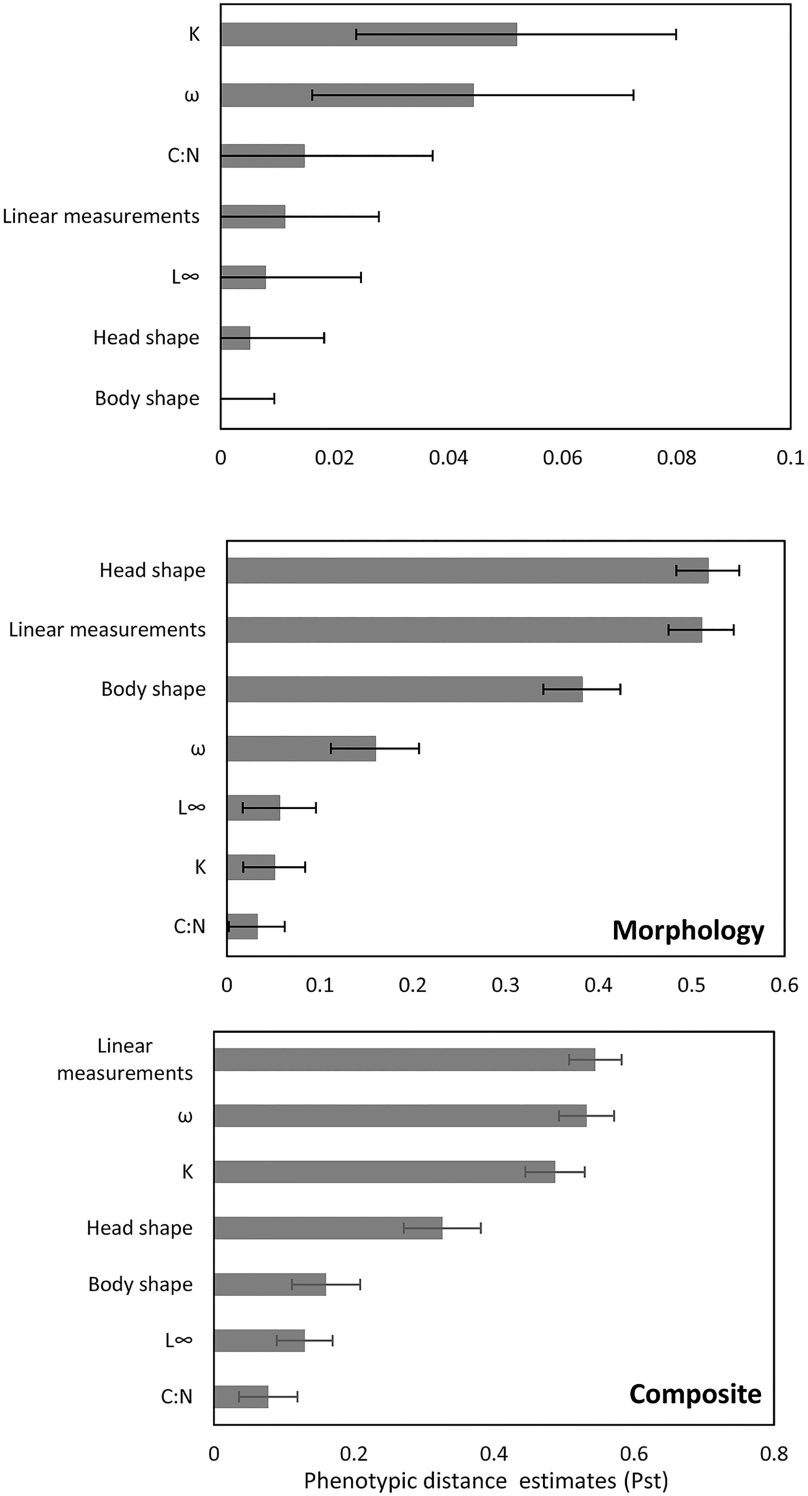
Global phenotypic trait divergence (*Pst*). Global phenotypic trait divergence (*Pst*) ± SE for individual variable for Lake Trout from Great Bear Lake based on groups established based on depth strata, morphological data, and composite data.

Great Bear Lake displayed high phenotypic divergence overall, whereas several phenotypic traits varied within depth, morphological and composite groupings (Fig 6). Life-history parameters (K and *ω*) displayed the most variation within groups organized by depth of capture, while *L*_∞_, head shape, and body shape varied the least (Fig 6). Phenotypic trait variations among the three groups identified by morphotypic data were influenced most by head shape, linear measurements, and body shape, while life-history parameters (K and *L*_∞_) and C:N ratio had the lowest values (Fig 6). Finally, phenotypic divergence among composite groups was driven by linear measurements, *ω*, and *K*, whereas *L*_∞_ and C:N varied the least (Fig 6).

## Discussion

Phenotypic divergence of Lake Trout between deep-water and shallow-water was weak in Great Bear Lake, with no distinct morph inhabiting the deep-water zone, but our composite dataset provided some evidence for adaptation to deep-water conditions (see [87–89] for other examples). Lake Trout diversification previously has been strongly associated with depth partitioning, and with Great Bear Lake having a maximum depth of 450 m, we expected intraspecific diversity to be organized along a depth axis. However, depth alone was not a major explanatory variable of Lake Trout diversity in Great Bear Lake, rather, an ecological continuum existed. Ecological clines, eco-spatial structure of diversity, where tensions between homogenizing and divergent evolutionary forces arise, can be components of the complex nature of phenotypic evolution across diverse and heterogeneous landscapes such as Great Bear Lake [90, 91]. Clines of intraspecific variation occur within freshwater ecosystems, where abiotic (e.g., temperature, light, and oxygen composition) and biotic variables (e.g., trophic resources, parasites, and predators) change in a predictable manner: 1) along a depth axis from shallow to deep, and 2) along a benthic-limnetic axis [92].

How often can clines of intraspecific variation exist without any geographic isolation? Isolation-by-distance is not an essential component in generating and maintaining intraspecific diversity within a lake, but variation can be facilitated by a number of variables, including barriers induced by differing habitat use [93, 94]. Large deep lakes, such as Great Bear Lake, are more likely to provide reproductive isolating barrier(s) through isolation by geographic distance and lake bathymetry than small lakes simply because of their size (but no information currently exists about Great Bear Lake spawning habitat and behavior) [17, 22–24, 95, 96]. Indeed, when examining a large lake that sustains high Lake Trout diversity (e.g., Lake Superior), depth was a more important axis of genetic divergence than genetic differences among morphs [22]. Overall, high levels of gene flow were inferred among Lake Trout groups in Great Bear Lake; only deep-water Lake Trout showed low levels of genetic differentiation when compared based on depth-at-capture (i.e., 51–150 m versus 0–20 and 21–50 m). Thus, Lake Trout in Great Bear Lake appeared vary slightly along the depth gradient but divergence also occurred along other niche axes (different degrees of omnivory along a weak benthic–pelagic gradient in shallow-water habitats) [27], i.e., diversification was not solely associated with depth.

Ecological opportunity associated with the depth gradient of Great Bear Lake may be promoting adaptive diversification, as evidenced in differences in a suite of ecological and morphological characteristics. Potential evidence of adaptation to deep- vs. shallow-water habitat was found in the relationship between C:N ratio and depth. Individuals captured in deep-water habitat showed a higher C:N ratio linked with a higher lipid content (i.e., adaptive or not) than trout caught in shallow-water, a characteristic generally associated with buoyancy [67, 69–71]. Variation in buoyancy of Lake Trout has been associated with adaptation to deep-water habitats observed across North American lakes [18, 19, 21, 23]. High fat content enhances buoyancy, enables diel vertical migration, and can improve foraging efficiency in deep-water habitat [15, 97–99]. Phenotypic divergence in linear measurements (e.g., fin lengths) were also displayed when groups were defined by composite variables, which also may be related to variation in swimming tactics and foraging strategies associated with deep- vs. shallow-water habitats. Each morphological characteristic (i.e., body shape *vs*. head shape *vs*. linear measurements) can have different degrees of plastic responses depending on the strength and duration of exposure to heterogeneous environments [42, 100–102]. Indeed, linear measurements have been demonstrated to be highly plastic and to vary in Great Bear Lake compared to body and head shape [25]. Finally, phenotypic divergence in life history was also observed in relation to depth, especially in back-calculated growth rates of juveniles and adults (*ω* and K, respectively), and were important parameters in the composite grouping that identified the deep-water individuals.

Diversifying forces are generally caused by a mismatch between a population’s niche-related traits and newly encountered ecological conditions [26, 39, 92]. Although isotopic niche overlap was evident among groups, Lake Trout caught in the deep zone of Great Bear Lake seemed to have a higher trophic level than their shallow-water counterparts. Higher trophic level might result from an increase in piscivory (possibly including cannibalism due to its high observed level within the system) or a different isotopic enrichment signal linked with depth [27, 32, 103]. Deep-water morphs of Cisco (*Coregonus artedi*), deep-water Sculpins (*Myoxocephalus quadricornis and Cottus cognatus)*, and *Mysis* inhabit Great Bear Lake [104]; together with juvenile and adult Lake Trout; these represent the differing foraging opportunities for deep-water Lake Trout. In the shallow-water habitat, however, more abundant and diverse prey sources (e.g., littoral fish, macroinvertebrates) are available to Lake Trout as foraging opportunities and are suspected to reflect higher productivity and diversity than in the deep water [27, 32, 40]. The trophic niche associated with the deep-water habitat of Great Bear Lake may not provide many opportunities for further specialization or diversification as compared to other North American lakes [105]. Great Bear Lake has extremely low productivity [38], especially in the deep-water habitat, once described as a “biological wasteland” by Miller [106]. The differences between adaptive landscapes surrounding a species might be too weak to prevent a major diet divergence despite our assumption that shallow- and deep-water habitats are different. Similarly, low productivity combined with a lack of unique spatial and temporally distributed energy sources would be consistent with the level of intraspecific diversity expressed within the shallow-water habitat, where three generalist morphs coexist without strong evidence that this intraspecific diversity is resource-induced [27]. Benthic primary and secondary production is known to be an important energy source in Arctic lakes, which in many Arctic lakes is focused in littoral areas [107]. Most cases of polymorphism in freshwater fishes have been described as a function of the discretness between habitats and foraging opportunities (e.g., shallow-profundal or littoral-benthic: [108–110], but fine spatial scales can also influence functional diversity [9, 22, 45, 89, 111–113].

Why is phenotypic divergence between shallow- and deep-water Lake Trout in Great Bear Lake lower than expected is unknown, especially when the potential for phenotypic variation seems high (e.g., [45], and given that depth partitioning is associated with Lake Trout diversity elsewhere). Several reasons may explain our observations: 1) high gene flow (discussed above), 2) divergent selection might be relative weak (discussed above), 3) more evolutionary time might be necessary [114], and 4) our sample size may have been too small.

Continuous reaction norms of phenotypic plasticity along a gradient often reveal more complex patterns than between two discrete environments [115]. Although Lake Trout can be found across all depths, phenotypic diversity was inconsistently expressed along the depth gradient in Great Bear Lake. Intraspecific diversity of Lake Trout from Great Bear Lake, from high diversity in shallow-water to low diversity in deep-water habitats, could reflect unequal selection intensity along the depth continuum, thereby resulting in the expression of phenotypic plasticity across a landscape [90]. In Great Bear Lake, the deep-water environment may be more homogenous and predictable than the shallow-water environment, and thus phenotypic diversity decreased with increasing depth [116–119]. Outcomes depend, in part, on costs and developmental limitations to plasticity, influencing the expression of plasticity as a response to particular ecological conditions, which can lead to dramatic fitness benefits, compared with a lack of a plastic response [119–122].

Given that variation in complexity of freshwater environments has dramatic consequences for divergence [92, 123], variation in the complexity in Great Bear Lake (i.e., shallow being more complex than deep) [40], may explain the observed dichotomy in the expression of intraspecific phenotypic diversity between shallow- vs. deep-water habitats. If phenotypic variation is not genetic but strictly environmental in origin, observed intraspecific differences might ultimately be trivial in terms of the ongoing process of adaptation [123]. However, if variation has a genetic component whose expression is triggered by the environment, then phenotypes can be refined by selection. Complete divergence becomes possible, especially if shifts become more extreme along the same environmental dimension or across multi-dimensions [124–126].

The final question regarding the Lake Trout of Great Bear Lake is whether the gradient of incipient divergence we observed has had enough time to fully differentiate into evolutionary units or if a stable “intermediate” pattern exists between monomorphic and polymorphic [18, 127]. The level of niche divergence associated with depth within this system was lower than in other lakes in North America, with such patterns usually strong enough to be determined even within small sample sizes [23]. The low F_ST_ values we observed may indicate: 1) a short time since inception of divergence because phenotypic variations were accumulated by deep-water Lake Trout over ~350 generations [34], 2) high gene flow that prevents accumulation of adaptations [128, 129], or 3) large effective population sizes, rendering drift negligible in promoting differentiation among groups. Whether the observed variation with depth reported here is “stable” or is an initial divergent step that with enough time will fully differentiate evolutionary units is unknown, but it is inherently hard to make any conclusions from studies such as ours as to what a “real” initial stage of divergence is [130]. Nonetheless, when looked at patterns described in salmonid species inhabiting other post-glacial colonized ecosystems, including small lakes, the formation of highly distinct sympatric populations is often rapid [131–133]. Thus, the novelty of Great Bear Lake, herein, relies in its size and bathymetric complexity of this system but minimal apparent cline of diversification, despite the propensity of salmonids to vary rapidly, especially with depth.

One of the points of contention about understanding mechanisms driving intraspecific ecological divergence is the extent of variability displayed among species and systems [124]. Intraspecific diversity represents multiple evolutionary outcomes that can either promote or constrain progress toward ecological speciation, and may not always result in easily identifiable differentiation stages [92, 116, 119]. Cryptic population structures are difficult to uncover and more common than previously thought [87, 89, 134, 135]. Great Bear Lake deep-water Lake Trout could be a cryptic example of divergence, because individuals displayed variation in association with the profundal habitat, whereas their morphological differentiation was not defined sufficiently to be identified as a distinct evolutionary unit. The debate around diversification sequence, i.e., what diverges first, morphology or ecology, highlights the mosaic nature of speciation [125] and reminds us how difficult it often is to classify and disentangle divergence events.

## Conclusion

Rates of speciation for some freshwater fishes are among the highest known for vertebrates, but spatial and temporal distribution of energy and physical habitats, limits the number of species or morphs that can co-exist [92, 136]. However, we do not understand the extent and relative importance of different variables that constrain or promote diversity [137]. Ecological opportunity cannot be the entire story of diversification, because the presence of ecological opportunity has not always led to adaptive radiation, which raises the question whether adaptive radiation can occur in the absence of ecological opportunity [3]. In Great Bear Lake, intraspecific diversity of Lake Trout, with its high phenotypic variation not strongly associated with either horizontal or vertical ecological partitioning axes [27], does not represent the usual pattern of divergence observed within Lake Trout elsewhere or salmonids in general. This lack of correspondence to the ecological theory of adaptive radiation [36, 138, 139] joins examples across a variety of taxa that counter the long-standing hypothesis that specialized morphology corresponds to a specialist diet. Possibly, such situations might be more frequent than previously thought [140]. Multiple niche axes along different environmental gradients appear to structure Lake Trout diversity within Great Bear Lake. If multiple selection axes exist in Great Bear Lake, the question then arises as to whether they favor divergence or counteract each other’s influences? Opportunities to study examples when phenotypic variation is high (fish are really plastic, see e.g., [31, 45, 114]) are useful in understanding the origin and fate of incipient stages of speciation. The ambiguity surrounding the mechanism(s) driving divergence in Lake Trout of Great Bear Lake should be seen as part of the highly variable nature of ecological opportunity and divergent natural selection itself [100, 124].

## Supporting information

S1 Table Mean log-likelihood values (LnP[K]) for different hypothesized numbers of genetic populations (K) of Lake Trout in Great Bear Lake. Also shown is the mean value of ΔK, the *ad hoc* statistic of Evanno et al. [63] used to summarize the second-order rate of change in LnP(K). The bold values represent the most likely number of genetic groups for each statistic for each clustering scenario. NA = ΔK cannot be calculated for these values of K.
(DOCX)

S2 Table Length-age models within three depth strata strata (Depth) in Great Bear Lake. Length-age models for Lake Trout captured within three depth strata (Depth) in Great Bear Lake. Each model is specified to compare growth among Lake Trout at different depths of capture (Depth), and varying growth parameters (*t*_0_, *L*_∞_,*K*), along with the number of parameters (*df*), log-likelihood (logLik), Akaike Information Criterion (*AIC*), Akaike difference (Δ_*i*_), and Akaike weight (*w*_*i*_).
(DOCX)

S3 Table Growth parameter estimates within three depth strata in Great Bear Lake. Growth parameter estimates for Lake Trout captured within three depth strata in Great Bear Lake (SE = standard error; LL = lower 95% confidence limit; UL = upper 95% confidence limit).
(DOCX)

S4 Table Length-age models for three Lake Trout morphs captured in Great Bear Lake, Northwest Territories. Length-age models for three Lake Trout morphs (Morph) captured in Great Bear Lake, Northwest Territories. Each model is specified to compare growth among Lake Trout morphs (Morph) and varying growth parameters (*t*_0_, *L*_∞_,*K*), along with the number of parameters (df), log-likelihood (logLik), Akaike Information Criterion (AIC), Akaike difference (Δ_i_), and Akaike weight (*w*_i_).
(DOCX)

S5 Table Growth parameter estimates for three Lake Trout morphs captured in Great Bear Lake. (SE = standard error; LL = lower 95% confidence limit; UL = upper 95% confidence limit).
(DOCX)

S6 Table Length-age models for four Lake Trout composite groups captured in Great Bear Lake. Each model is specified to compare growth among Lake Trout composite groups and varying growth parameters (*t*_0_, *L*_∞_,*K*), along with the number of parameters (df), log-likelihood (logLik), Akaike Information Criterion (AIC), Akaike difference (Δ_i_), and Akaike weight (*w*_i_).
(DOCX)

S7 Table Growth parameter estimates for four lake trout composite groups captured in Great Bear Lake. (SE = standard error; LL = lower 95% confidence limit; UL = upper 95% confidence limit).
(DOCX)

S1 Fig The four shallow-water morphotypes of Lake Trout from Great Bear Lake. (DOCX)

S2 Fig Catch-per-unit-effort of adult Lake Trout captured among depth strata in Great Bear Lake. Catch-per-unit-effort (median and quartiles) of adult Lake Trout captured among depth strata in Great Bear Lake. No CPUE differences were found among the three depth strata for catch of adult Lake Trout (F_2,23_ = 0.12, p = 0.89).
(DOCX)

S3 Fig Systematic design of each grouping procedure used in this study, based on depth strata, morphology, and composite variables. Each grouping method used a different suite of variables to assign individuals to a group. After groups were identified, subsequent analyses followed for patterns in morphology, genetics, isotopes, and life-history parameters among groups with each procedure. See text for more detail.
(DOCX)

S4 Fig Hierarchical clusters of Lake Trout individuals sampled in Dease Arm (0–150 m) overlaid on the first two principal component axes (PCA) using FactoMineR [46], in A) based on morphological grouping and in B) based on composite grouping. Grouping by the morphological procedure: Morph 1 = red circle, Morph 2 = green circle, and Morph 3 = black circle, and by the composite procedure: Comp 1 = green circle, Comp 2 = blue circle, Comp 3 = black circle, and Comp 4/deep-water individuals = red circle. PCA variables are defined as follow: PC1BS and PC2BS first two-axis PCA scores of body shape from landmarks, PC1HS and PC2HS = first two-axis PCA scores of head shape from semi-landmarks, PC1LM and PC2LM = first two-axis PCA scores of linear measurements, PC1GEN and PC2GEN = first two-axis PCA scores of genetic data, Delta C 13 = δ^13^C, Delta N 15 = δ^15^N, K = von Bertalanffy growth parameter (adult), *ω* = juvenile growth rate, *L*_∞_ = maximum adult length, depth-at-capture.
(DOCX)

S5 Fig CVA of Lake Trout linear measurements, body shape, and head shape. Groups were identified by FactoMineR [46] based on morphological and composite *group assignment*. Each group is also outlined by a 68.3% confidence ellipse. For the depth procedure, groups are represented as follows: open circle = 0–20 m, light grey square = 21–50 m, and black diamond = 51–150 m. For morphological procedure, groups are represented as follows: Morph 1 = white, Morph 2 = black, and Morph 3 = light grey. For composite assignments, groups are represented as follows: x = Comp 1, ⋆ = Comp 2, star = Comp 3, and triangle = Comp 4 (deep-water individuals).
(DOCX)
