# Supplementary material for: From top to bottom: Do Lake Trout diversify along a depth gradient in Great Bear Lake, NT, Canada?
Source: PLoS One. 2018 Mar 22;13(3):e0193925. doi: 10.1371/journal.pone.0193925 (PMC5863968; doi:10.1371/journal.pone.0193925)
Supplement: S1 Table — Also shown is the mean value of ΔK, the ad hoc statistic of Evanno et al. [63] used to summarize the second-order rate of change in LnP(K). The bold values represent the most likely number of genetic groups for each statistic for each clustering scenario. NA = ΔK cannot be calculated for these values of K. (DOCX) [file pone.0193925.s001.docx]

S1 Table. Mean log-likelihood values (LnP[K]) for different hypothesized numbers of genetic populations (K) of Lake Trout in Great Bear Lake. Also shown is the mean value of ΔK, the *ad hoc* statistic of Evanno, Regnaut (62) used to summarize the second-order rate of change in LnP(K). The bold values represent the most likely number of genetic groups for each statistic for each clustering scenario. NA = ΔK cannot be calculated for these values of K.

| **# K** | **Depth** | | **Morphology** | | **Composite** | |
| --- | --- | --- | --- | --- | --- | --- |
|  | Mean LnP(K) | *∆*K | Mean LnP(K) | *∆*K | Mean LnP(K) | *∆*K |
| **1** | **-21724** | — | **-12512** | — | **-10285** | — |
| **2** | -21735 | 0.20 | -12673 | 0.24 | -10504 | 0.36 |
| **3** | -21737 | **1.57** | -12873 | 0.01 | -10609 | 0.11 |
| **4** | -21927 | 0.98 | -13068 | 0.01 | -10689 | **0.61** |
| **5** | -22404 | 0.43 | -13267 | 0.72 | -10944 | 0.55 |
| **6** | -22630 | 0.44 | -13749 | **0.91** | -10888 | 0.24 |
| **7** | -23143 | 0.33 | -13694 | 0.56 | -10719 | 0.31 |
| **8** | -23922 | 0.68 | -13952 | 0.15 | -10649 | 0.37 |
| **9** | -23501 | 0.52 | -14113 | 0.42 | -10681 | 0.36 |
| **10** | -24079 | NA | -13773 | NA | -10614 | NA |
