# Supplementary material for: From top to bottom: Do Lake Trout diversify along a depth gradient in Great Bear Lake, NT, Canada?
Source: PLoS One. 2018 Mar 22;13(3):e0193925. doi: 10.1371/journal.pone.0193925 (PMC5863968; doi:10.1371/journal.pone.0193925)
Supplement: S2 Table — Length-age models for Lake Trout captured within three depth strata (Depth) in Great Bear Lake. Each model is specified to compare growth among Lake Trout at different depths of capture (Depth), and varying growth parameters (t0, L∞,K), along with the number of parameters (df), log-likelihood (logLik), Akaike Information Criterion (AIC), Akaike difference (Δi), and Akaike weight (wi). (DOCX) [file pone.0193925.s002.docx]

S2 Table. Length-age models for Lake Trout captured within three depth strata (Depth) in Great Bear Lake. Each model is specified to compare growth among Lake Trout at different depths of capture (Depth), and varying growth parameters (*t*_0_, *L*_∞_,*K*), along with the number of parameters (*df*), log-likelihood (logLik), Akaike Information Criterion (*AIC*), Akaike difference (Δ*_i_*), and Akaike weight (*w_i_*).

| Model | *df* | logLik | *AIC* | Δ*_i_* | *e*^(−0.5×Δi)^ | *w_i_* |
| --- | --- | --- | --- | --- | --- | --- |
| Depth(*K*) | 12 | −15238.32 | 30500.64 | 0.00 | 1.000000000 | 0.35 |
| Depth(*t*_0_,*K*) | 14 | −15236.45 | 30500.90 | 0.26 | 0.878095431 | 0.30 |
| Depth(*t*_0_, *L*_∞_,*K*) | 16 | −15234.96 | 30501.92 | 1.28 | 0.527292424 | 0.18 |
| Depth(*L*_∞_,*K*) | 14 | −15237.35 | 30502.70 | 2.06 | 0.357006961 | 0.12 |
| Depth(*L*_∞_) | 12 | −15241.15 | 30506.30 | 5.66 | 0.059012854 | 0.02 |
| (*t*_0_, *L*_∞_,*K*) | 10 | −15243.63 | 30507.26 | 6.62 | 0.036516174 | 0.01 |
| Depth(*t*_0_) | 12 | −15242.38 | 30508.76 | 8.12 | 0.017249019 | 0.01 |
| Depth(*t*_0_, *L*_∞_) | 14 | −15240.45 | 30508.90 | 8.26 | 0.016082879 | 0.01 |
