# Supplementary material for: From top to bottom: Do Lake Trout diversify along a depth gradient in Great Bear Lake, NT, Canada?
Source: PLoS One. 2018 Mar 22;13(3):e0193925. doi: 10.1371/journal.pone.0193925 (PMC5863968; doi:10.1371/journal.pone.0193925)
Supplement: S3 Table — Growth parameter estimates for Lake Trout captured within three depth strata in Great Bear Lake (SE = standard error; LL = lower 95% confidence limit; UL = upper 95% confidence limit). (DOCX) [file pone.0193925.s003.docx]

S3 Table. Growth parameter estimates for Lake Trout captured within three depth strata in Great Bear Lake (SE = standard error; LL = lower 95% confidence limit; UL = upper 95% confidence limit).

| Parameter | Depth | Estimate | SE | LL | UL |
| --- | --- | --- | --- | --- | --- |
| *t*_0_ | 0–20 m | −1.13 | 0.13 | −1.38 | −0.88 |
|  | 21–50 m | −1.50 | 0.11 | −1.72 | −1.28 |
|  | 51-150 m | −1.25 | 0.17 | −1.58 | −0.92 |
| *L*_∞_ | 0–20 m | 852.93 | 20.66 | 812.49 | 893.38 |
|  | 21–50 m | 882.71 | 18.66 | 846.18 | 919.24 |
|  | 51-150 m | 826.47 | 27.22 | 773.16 | 879.78 |
| *K* | 0–20 m | 0.081 | 0.0033 | 0.074 | 0.087 |
|  | 21–50 m | 0.066 | 0.0029 | 0.060 | 0.072 |
|  | 51-150 m | 0.070 | 0.0044 | 0.062 | 0.079 |
| *L*_∞_ | 0–20 m | 852.66 | 20.85 | 811.83 | 893.48 |
|  | 21–50 m | 882.86 | 18.84 | 845.97 | 919.76 |
|  | 51-150 m | 826.31 | 27.53 | 772.41 | 880.21 |
| *L*_0_ | 0–20 m | 60.90 | 4.70 | 51.71 | 70.09 |
|  | 21–50 m | 72.43 | 4.13 | 64.34 | 80.52 |
|  | 51-150 m | 63.7981 | 6.06 | 51.93 | 75.67 |
| *ω* | 0–20 m | 67.19 | 2.45 | 62.40 | 71.98 |
|  | 21–50 m | 57.00 | 2.16 | 52.78 | 61.23 |
|  | 51-150 m | 57.63 | 3.17 | 51.43 | 63.83 |
