# Supplementary material for: From top to bottom: Do Lake Trout diversify along a depth gradient in Great Bear Lake, NT, Canada?
Source: PLoS One. 2018 Mar 22;13(3):e0193925. doi: 10.1371/journal.pone.0193925 (PMC5863968; doi:10.1371/journal.pone.0193925)
Supplement: S4 Table — Length-age models for three Lake Trout morphs (Morph) captured in Great Bear Lake, Northwest Territories. Each model is specified to compare growth among Lake Trout morphs (Morph) and varying growth parameters (t0, L∞,K), along with the number of parameters (df), log-likelihood (logLik), Akaike Information Criterion (AIC), Akaike difference (Δi), and Akaike weight (wi). (DOCX) [file pone.0193925.s004.docx]

S4 Table. Length-age models for three Lake Trout morphs (Morph) captured in Great Bear Lake, Northwest Territories. Each model is specified to compare growth among Lake Trout morphs (Morph) and varying growth parameters (*t*_0_, *L*_∞_,*K*), along with the number of parameters (df), log-likelihood (logLik), Akaike Information Criterion (AIC), Akaike difference (Δ_i_), and Akaike weight (*w*_i_).

| Model | *df* | logLik | *AIC* | Δ*_i_* | *e*^(−0.5×Δi)^ | *w_i_* |
| --- | --- | --- | --- | --- | --- | --- |
| Morph(*t*_0_, *L*_∞_,*K*) | 16 | −15220.93 | 30473.86 | 0.00 | 1.00 | 0.86 |
| Morph(*t*_0_,*K*) | 14 | −15225.62 | 30479.24 | 5.38 | 0.068 | 0.06 |
| Morph(*L*_∞_) | 12 | −15228.06 | 30480.12 | 6.26 | 0.044 | 0.04 |
| Morph(*t*_0_, *L*_∞_) | 14 | −15226.24 | 30480.48 | 6.62 | 0.037 | 0.03 |
| Morph(*L*_∞_,*K*) | 14 | −15226.86 | 30481.72 | 7.86 | 0.020 | 0.02 |
| Morph(*K*) | 12 | −15235.25 | 30494.50 | 20.64 | ≤ 0.01 | 0.00 |
| (*t*_0_, *L*_∞_,*K*) | 10 | −15243.63 | 30507.26 | 33.40 | ≤ 0.01 | 0.00 |
| Morph(*t*_0_) | 12 | −15242.20 | 30508.40 | 34.54 | ≤ 0.01 | 0.00 |
