# Supplementary material for: From top to bottom: Do Lake Trout diversify along a depth gradient in Great Bear Lake, NT, Canada?
Source: PLoS One. 2018 Mar 22;13(3):e0193925. doi: 10.1371/journal.pone.0193925 (PMC5863968; doi:10.1371/journal.pone.0193925)
Supplement: S5 Table — (SE = standard error; LL = lower 95% confidence limit; UL = upper 95% confidence limit). (DOCX) [file pone.0193925.s005.docx]

S5 Table. Growth parameter estimates for three Lake Trout morphs captured in Great Bear Lake (SE = standard error; LL = lower 95% confidence limit; UL = upper 95% confidence limit).

| Parameter | Morph | Estimate | SE | LL | UL |
| --- | --- | --- | --- | --- | --- |
| *t*_0_ | Morph 1 | −1.55 | 0.12 | −1.79 | −1.31 |
|  | Morph 2 | −1.04 | 0.11 | −1.25 | −0.83 |
|  | Morph 3 | −1.60 | 0.18 | −1.95 | −1.24 |
| *L*_∞_ | Morph 1 | 862.91 | 20.08 | 823.60 | 902.23 |
|  | Morph 2 | 887.07 | 17.81 | 852.19 | 921.95 |
|  | Morph 3 | 783.65 | 28.58 | 727.68 | 839.62 |
| *K* | Morph 1 | 0.07 | 0.0033 | 0.062 | 0.074 |
|  | Morph 2 | 0.08 | 0.0029 | 0.073 | 0.084 |
|  | Morph 3 | 0.06 | 0.0047 | 0.053 | 0.071 |
| *L*_∞_ | Morph 1 | 864.07 | 20.23 | 824.45 | 903.68 |
|  | Morph 2 | 886.52 | 17.97 | 851.34 | 921.70 |
|  | Morph 3 | 784.00 | 28.79 | 727.63 | 840.37 |
| *L*_0_ | Morph 1 | 75.57 | 4.49 | 66.78 | 84.36 |
|  | Morph 2 | 58.35 | 3.99 | 50.54 | 66.16 |
|  | Morph 3 | 70.56 | 6.48 | 57.88 | 83.25 |
| *ω* | Morph 1 | 57.43 | 2.24 | 53.04 | 61.82 |
|  | Morph 2 | 67.72 | 1.99 | 63.84 | 71.61 |
|  | Morph 3 | 47.98 | 3.25 | 41.61 | 54.36 |
