# Supplementary material for: From top to bottom: Do Lake Trout diversify along a depth gradient in Great Bear Lake, NT, Canada?
Source: PLoS One. 2018 Mar 22;13(3):e0193925. doi: 10.1371/journal.pone.0193925 (PMC5863968; doi:10.1371/journal.pone.0193925)
Supplement: S6 Table — Each model is specified to compare growth among Lake Trout composite groups and varying growth parameters (t0, L∞,K), along with the number of parameters (df), log-likelihood (logLik), Akaike Information Criterion (AIC), Akaike difference (Δi), and Akaike weight (wi). (DOCX) [file pone.0193925.s006.docx]

S6 Table. Length-age models for four Lake Trout composite groups captured in Great Bear Lake. Each model is specified to compare growth among Lake Trout composite groups and varying growth parameters (*t*_0_, *L*_∞_,*K*), along with the number of parameters (df), log-likelihood (logLik), Akaike Information Criterion (AIC), Akaike difference (Δ_i_), and Akaike weight (*w*_i_).

| Model | *df* | logLik | *AIC* | Δ*_i_* | *e*^(−0.5×Δi)^ | *w_i_* |
| --- | --- | --- | --- | --- | --- | --- |
| Composite group (*t*_0_,*L*_∞_,*K*) | 19 | −12784.61 | 25607.22 | 0.00 | 1.00 | 1.00 |
| Composite group (*L*_∞_,*K*) | 16 | −12795.73 | 25623.46 | 16.24 | ≤ 0.01 | 0.00 |
| Composite group (*t*_0_,*K*) | 16 | −12798.68 | 25629.36 | 22.14 | ≤ 0.01 | 0.00 |
| Composite group (*K*) | 13 | −12808.63 | 25643.26 | 36.04 | ≤ 0.01 | 0.00 |
| Composite group (*t*_0_, *L*_∞_) | 16 | −12830.17 | 25692.34 | 85.12 | ≤ 0.01 | 0.00 |
| Composite group (*L*_∞_) | 13 | −12835.35 | 25696.70 | 89.48 | ≤ 0.01 | 0.00 |
| Composite group (*t*_0_) | 13 | −12849.32 | 25724.64 | 117.42 | ≤ 0.01 | 0.00 |
| (t0,*L*_∞_,*K*) | 10 | −12857.95 | 25735.90 | 128.68 | ≤ 0.01 | 0.00 |
