# Supplementary material for: From top to bottom: Do Lake Trout diversify along a depth gradient in Great Bear Lake, NT, Canada?
Source: PLoS One. 2018 Mar 22;13(3):e0193925. doi: 10.1371/journal.pone.0193925 (PMC5863968; doi:10.1371/journal.pone.0193925)
Supplement: S7 Table — (SE = standard error; LL = lower 95% confidence limit; UL = upper 95% confidence limit). (DOCX) [file pone.0193925.s007.docx]

S7 Table. Growth parameter estimates for four lake trout composite groups captured in Great Bear Lake (SE = standard error; LL = lower 95% confidence limit; UL = upper 95% confidence limit).

| Parameter | Composite group | Estimate | SE | LL | UL |
| --- | --- | --- | --- | --- | --- |
| *t*_0_ | Comp 1 | −1.66 | 0.20 | −2.06 | −1.26 |
|  | Comp 2 | −1.50 | 0.11 | −1.72 | −1.27 |
|  | Comp 3 | −0.41 | 0.20 | −0.81 | −0.01 |
|  | Comp 4 | −1.31 | 0.17 | −1.64 | −0.99 |
| *L*_∞_ | Comp 1 | 772.94 | 30.58 | 713.10 | 832.78 |
|  | Comp 2 | 935.14 | 18.07 | 899.77 | 970.51 |
|  | Comp 3 | 788.27 | 31.52 | 726.58 | 849.96 |
|  | Comp 4 | 834.13 | 25.99 | 783.27 | 885.00 |
| *K* | Comp 1 | 0.063 | 0.0037 | 0.06 | 0.07 |
|  | Comp 2 | 0.063 | 0.0021 | 0.06 | 0.07 |
|  | Comp 3 | 0.12 | 0.0040 | 0.11 | 0.12 |
|  | Comp 4 | 0.068 | 0.0032 | 0.06 | 0.07 |
| *L*_∞_ | Comp 1 | 774.59 | 31.04 | 713.75 | 835.42 |
|  | Comp 2 | 935.01 | 18.31 | 899.14 | 970.89 |
|  | Comp 3 | 786.58 | 31.98 | 723.90 | 849.25 |
|  | Comp 4 | 832.26 | 26.40 | 780.51 | 884.02 |
| *L*_0_ | Comp 1 | 71.22 | 7.24 | 57.04 | 85.41 |
|  | Comp 2 | 74.59 | 4.14 | 66.47 | 82.71 |
|  | Comp 3 | 33.93 | 7.80 | 18.65 | 49.22 |
|  | Comp 4 | 66.81 | 6.05 | 54.95 | 78.68 |
| *ω* | Comp 1 | 47.71 | 2.70 | 42.42 | 53.00 |
|  | Comp 2 | 57.96 | 1.55 | 54.93 | 60.99 |
|  | Comp 3 | 57.96 | 1.55 | 54.93 | 60.99 |
|  | Comp 4 | 56.63 | 2.27 | 52.19 | 61.08 |
