# Supplementary material for: From top to bottom: Do Lake Trout diversify along a depth gradient in Great Bear Lake, NT, Canada?
Source: PLoS One. 2018 Mar 22;13(3):e0193925. doi: 10.1371/journal.pone.0193925 (PMC5863968; doi:10.1371/journal.pone.0193925)
Supplement: S1 Fig — (DOCX) [file pone.0193925.s008.docx]

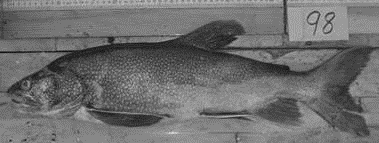

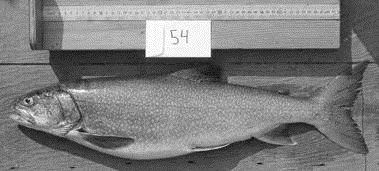

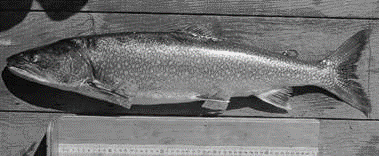

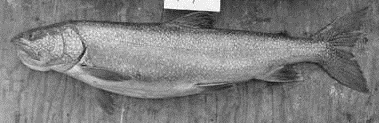


Morph 1

Morph 3

Morph 2

Morph 4

S1 Fig. The four shallow-water morphotypes of Lake Trout from Great Bear Lake identified in Chavarie et al. (2013, 2015, 2016a, 2016b).
