# Supplementary material for: From top to bottom: Do Lake Trout diversify along a depth gradient in Great Bear Lake, NT, Canada?
Source: PLoS One. 2018 Mar 22;13(3):e0193925. doi: 10.1371/journal.pone.0193925 (PMC5863968; doi:10.1371/journal.pone.0193925)
Supplement: S2 Fig — Catch-per-unit-effort (median and quartiles) of adult Lake Trout captured among depth strata in Great Bear Lake. No CPUE differences were found among the three depth strata for catch of adult Lake Trout (F2,23 = 0.12, p = 0.89). (DOCX) [file pone.0193925.s009.docx]

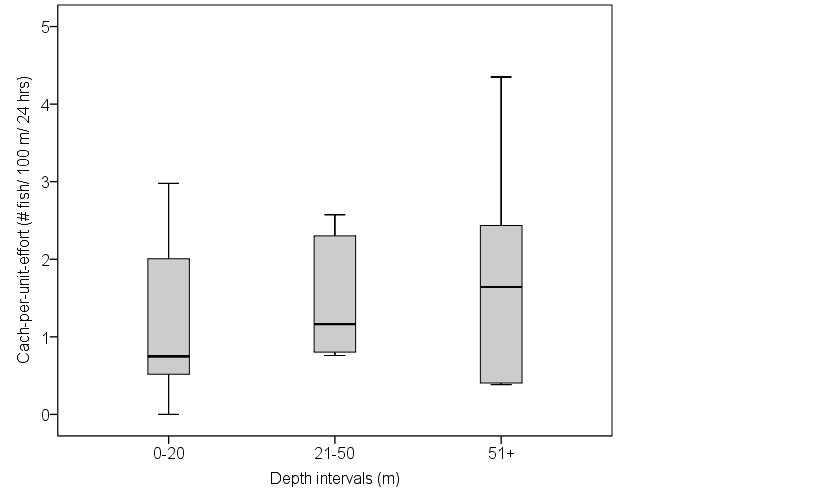


S2 Fig. Catch-per-unit-effort (median and quartiles) of adult Lake Trout captured among depth strata in Great Bear Lake. No CPUE differences were found among the three depth strata for catch of adult Lake Trout (F_2,23_ = 0.12, p= 0.89).
