# Supplementary material for: From top to bottom: Do Lake Trout diversify along a depth gradient in Great Bear Lake, NT, Canada?
Source: PLoS One. 2018 Mar 22;13(3):e0193925. doi: 10.1371/journal.pone.0193925 (PMC5863968; doi:10.1371/journal.pone.0193925)
Supplement: S3 Fig — Each grouping method used a different suite of variables to assign individuals to a group. After groups were identified, subsequent analyses followed for patterns in morphology, genetics, isotopes, and life-history parameters among groups with each procedure. See text for more detail. (DOCX) [file pone.0193925.s010.docx]

**Depth grouping procedure**

**Morphology grouping procedure**

**Composite grouping procedure**

**Variables:**

Depth-at-capture (categorical):

0-20, 21-50, 51-150 m

**Variables:**

PC1 Body shape

PC1 Head Shape

PC1 Linear measurements

**Variables:**

Depth-at-capture (continuous)

PC1 and PC2 Body shape

PC1 and PC2 Head Shape

PC1 and PC2 Linear measurements

PC1 and PC2 Genetic

δ^13^C and δ^15^N ‰

K, *ω,* and *L*_∞_

**Characteristics among groups within each grouping procedure:**

- Morphology: PCAs defined in “Variables” with grouping assignments for each individuals + CVAs
- Genetic: Structure + Genetic variation (N_A_, *H*_O_, *H*_E_, A_R_, PA_R_, F_IS_) + Pairwise F_ST_
- Isotopes: Isotopic niche
- Life-history: Life-history variation (*t*_0,_ *L*_∞,_ *K, L*_∞,_ *L*_0,_ *ω)*
- Phenotypic divergence : Global phenotypic trait divergence (Pst)
- variation (*t*_0,_ *L*_∞,_ *K, L*_∞,_ *L*_0,_ *ω)*
- Phenotypic divergence : Global phenotypic trait divergence (Pst)
- (*t*_0,_ *L*_∞,_ *K, L*_∞,_ *L*_0,_ *ω)*
- Phenotypic divergence : Global phenotypic trait divergence (Pst)

S3 Fig. Systematic design of each grouping procedure used in this study, based on depth strata, morphology, and composite variables. Each grouping method used a different suite of variables to assign individuals to a group. After groups were identified, subsequent analyses followed for patterns in morphology, genetics, isotopes, and life-history parameters among groups with each procedure. See text for more detail.
