# Supplementary material for: From top to bottom: Do Lake Trout diversify along a depth gradient in Great Bear Lake, NT, Canada?
Source: PLoS One. 2018 Mar 22;13(3):e0193925. doi: 10.1371/journal.pone.0193925 (PMC5863968; doi:10.1371/journal.pone.0193925)
Supplement: S4 Fig — Grouping by the morphological procedure: Morph 1 = red circle, Morph 2 = green circle, and Morph 3 = black circle, and by the composite procedure: Comp 1 = green circle, Comp 2 = blue circle, Comp 3 = black circle, and Comp 4/deep-water individuals = red circle. PCA variables are defined as follow: PC1BS and PC2BS first two-axis PCA scores of body shape from landmarks, PC1HS and PC2HS = first two-axis PCA scores of head shape from semi-landmarks, PC1LM and PC2LM = first two-axis PCA scores of linear measurements, PC1GEN and PC2GEN = first two-axis PCA scores of genetic data, Delta C 13 = δ13C, Delta N 15 = δ15N, K = von Bertalanffy growth parameter (adult), ω = juvenile growth rate, L∞ = maximum adult length, depth-at-capture. (DOCX) [file pone.0193925.s011.docx]

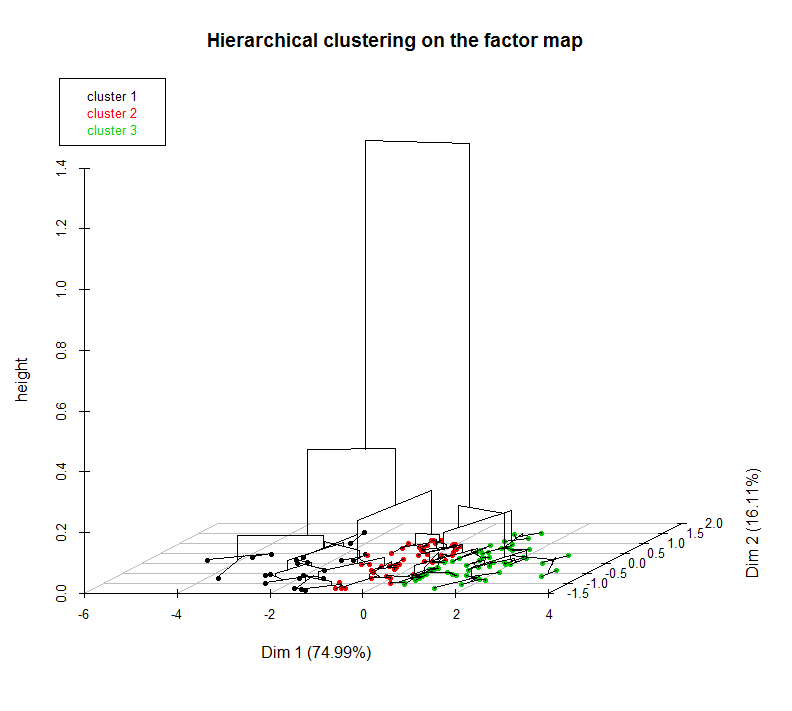


**Euclidean distance**

**PC1: 74.99%**

**PC2: 16.11%**

A)

**Euclidean distance**

**PC1: 24.64%**

**PC2: 18.18%**

B)

S4 Fig. Hierarchical clusters of Lake Trout individuals sampled in Dease Arm (0-150 m) overlaid on the first two principal component axes (PCA) using FactoMineR (45), in A) based on morphological grouping and in B) based on composite grouping. Grouping by the morphological procedure: Morph 1 = red circle, Morph 2= green circle, and Morph 3= black circle, and by the composite procedure: Comp 1= green circle, Comp 2 = blue circle, Comp 3 = black circle, and Comp 4/deep-water individuals=red circle. PCA variables are defined as follow: PC1BS and PC2BS first two-axis PCA scores of body shape from landmarks, PC1HS and PC2HS = first two-axis PCA scores of head shape from semi-landmarks, PC1LM and PC2LM = first two-axis PCA scores of linear measurements, PC1GEN and PC2GEN = first two-axis PCA scores of genetic data, Delta C 13= δ^13^C, Delta N 15= δ^15^N, K= von Bertalanffy growth parameter (adult), *ω* = juvenile growth rate, *L*_∞_ = maximum adult length, depth-at-capture.
