# Supplementary material for: From top to bottom: Do Lake Trout diversify along a depth gradient in Great Bear Lake, NT, Canada?
Source: PLoS One. 2018 Mar 22;13(3):e0193925. doi: 10.1371/journal.pone.0193925 (PMC5863968; doi:10.1371/journal.pone.0193925)
Supplement: S5 Fig — Groups were identified by FactoMineR [46] based on morphological and composite group assignment. Each group is also outlined by a 68.3% confidence ellipse. For the depth procedure, groups are represented as follows: open circle = 0–20 m, light grey square = 21–50 m, and black diamond = 51–150 m. For morphological procedure, groups are represented as follows: Morph 1 = white, Morph 2 = black, and Morph 3 = light grey. For composite assignments, groups are represented as follows: x = Comp 1, ⋆ = Comp 2, star = Comp 3, and triangle = Comp 4 (deep-water individuals). (DOCX) [file pone.0193925.s012.docx]

**Composite**

**Depth**

**Morphology**

**Linear measurements**

**Body Shape**

**Head Shape**

S5 Fig. CVA of Lake Trout linear measurements, body shape, and head shape. Groups were identified by FactoMineR (45) based on morphological and composite group assignment. Each group is also outlined by a 68.3% confidence ellipse. For the depth procedure, groups are represented as follows: open circle = 0-20 m, light grey square = 21-50 m, and black diamond = 51-150 m. For morphological procedure, groups are represented as follows: Morph 1 = white, Morph 2 = black, and Morph 3 = light grey. For composite assignments, groups are represented as follows: x = Comp 1, ⁎ = Comp 2, = Comp 3, and = Comp 4 (deep-water individuals).
